# Post-Coarctectomy Pseudoaneurysm with Recurrent Coarctation Treated with Open Surgery: A Comprehensive Literature Review and Case Report

**DOI:** 10.3390/jcm14165800

**Published:** 2025-08-16

**Authors:** Serena Arianna Cutolo, Diego Soto, Diletta Loschi, Annarita Santoro, Horatius Moldovan, Marian Broasca, Germano Melissano

**Affiliations:** 1 Division of Vascular Surgery, IRCCS San Raffaele Scientific Institute, Vita-Salute San Raffaele University, 20132 Milan, Italy; cutolo.serenaarianna@hsr.it (S.A.C.); d.soto@studenti.unisr.it (D.S.); loschi.diletta@hsr.it (D.L.); melissano.germano@hsr.it (G.M.); 2 Clinical Emergency Hospital Bucharest, “Carol Davila” University of Medicine and Pharmacy Bucharest, Calea Floreasca-8, Sector 1, 014461 Bucharest, Romania; h_moldovan@hotmail.ro (H.M.); marian.broasca@umfcd.ro (M.B.)

**Keywords:** aortic coarctation, pseudoaneurysm, thoracic aorta, thoracotomy, post-coarctation aneurysm

## Abstract

**Introduction:** Patch aortoplasty for aortic coarctation may have a high rate of late aneurysm and pseudoaneurysm formation, with an elevated risk of rupture and subsequent mortality when it occurs. **Case:** A 55-year-old male with irregular follow-up after patch aortoplasty for aortic coarctation 15 years ago, presented with a late post-coarctectomy aortic arch pseudoaneurysm and re-coarctation. Through a redo thoracotomy and under left heart bypass, the pseudoaneurysm and previous patch were partially excised, and reconstruction with interposition of a “Y” shaped Dacron graft was performed, in order to preserve the patency of the left subclavian artery. The postoperative course was uneventful, and at 6 months, the CT-angio control showed adequate graft patency. **Comprehensive Literature Review:** A comprehensive literature review with the primary focus on the different late complications after CoA repair (post-aortic coarctation aneurysms and recoarctation), and outcomes of open and endovascular approaches was performed. **Conclusions:** Open surgical repair for post-coarctectomy pseudoaneurysm is feasible with excellent results. However, due to the technical challenges, it may be performed with better results in high-volume aortic centers.

## 1. Introduction

Aortic coarctation (CoA) is characterized by a narrowing of the aorta, typically occurring near the ligamentum arteriosum, adjacent to the origin of the left subclavian artery (LSA) [1]. Both thoracic endovascular aortic repair (TEVAR) and open surgical repair (OSR) techniques have been described for CoA repair [2]. The surgical techniques for treating CoA are the subclavian flap, graft interposition and synthetic patch aortoplasty [3]. The latter aims to reduce restenosis rates by avoiding circumferential sutures, but its use is currently discouraged due to a high incidence of restenosis and late aneurysms and pseudoaneurysms (up to 51%) [4]. Regardless of the type of repair, lifelong surveillance is recommended [5].

Post-coarctectomy pseudoaneurysm formation has been more frequently reported following patch aortoplasty and is associated with a high incidence of spontaneous rupture (up to 31%) [6] and elevated mortality rates (78–100%) [6,7] when this occurs.

The present study includes a comprehensive literature review that discusses the different management options for late complication after CoA repair, including aneurysmal degeneration. It describes the outcomes of open versus endovascular approaches, and a real-life clinical case, that aims to illustrate the feasibility of OSR through a redo thoracotomy in a young and fit patient with inadequate anatomy for an endovascular approach in a high-volume aortic referral center.

## 2. Case Report Description

**Figure 1:**
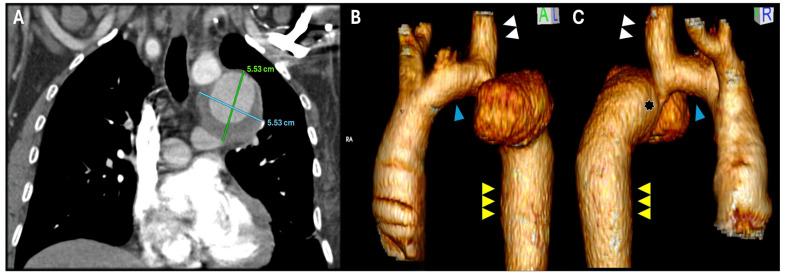
Preoperative CTA (**A**) and 3D reconstruction (**B**,**C**) showing the post-coarctectomy aneurysm on the distal segment of the aortic arch. Hypoplasia of the proximal aortic arch ((**B**,**C**), blue arrowhead), hypertrophy of the LSA ((**B**,**C**), white double arrowhead) and dilation of the distal thoracic aorta ((**B**,**C**), yellow triple arrowhead) can be identified. Re-coarctation signs are depicted ((**C**), asterisk) compatible with the preoperative pressure gradient.

A 55-year-old male with hypertension, dyslipidemia, and a bicuspid aortic valve underwent a patch aortoplasty for CoA in his country of origin at the age of 40, without regular follow-up. He presented at the emergency department of his native country hospital, reporting a history of mild exertional dyspnea and palpitations. At that time, a mild lower respiratory tract infection was diagnosed, and due to his surgical history, a computerized tomography angiography (CTA) was performed, showing a pseudoaneurysm (5.5 × 6 cm) near the origin of the LSA, associated with an aortic lumen narrowing at this level, compatible with a recurrence of the aortic coarctation (Figure 1A).

Based on these findings and the complexity of the case, the patient was referred to our hospital. Upon admission, the patient was asymptomatic. The blood pressure was 124/70 mmHg in the right arm, while in the lower limbs was 98/66 mmHg on the right and 104/65 mmHg on the left. Transthoracic echocardiography confirmed the presence of a bicuspid aortic valve (BAV) without functional abnormalities, a left ventricular ejection fraction (LVEF) of 63% and a transcoarctation gradient of 46 mmHg. Coronary angiography showed no relevant lesions and the other standard laboratory tests were within normal range.

Preoperative planning evidenced a barely sufficient proximal neck length of 18 mm (between the distal edge of the LCCA and the origin of the LSA) with a significant diameter mismatch between the distal aortic arch (18 mm in zone 2) and the distal thoracic aorta (35 mm in zone 3), associated with a dilated LSA (18 mm) (Figure 1B,C). According to these anatomical features, the recurrent coarctation and the patient’s age, an endovascular approach was discarded.

Based on the preoperative assessment, OSR was scheduled according to our center’s protocol for major open aortic surgery [8,9]. Intraoperative setup included double-lumen endobronchial tube, right radial and femoral arterial lines, somatosensory- and motor-evoked potentials monitoring (SMEP), transesophageal echocardiography and automated continuous cerebrospinal fluid (CSF) drainage with the LiquoGuard 3.0 system (LiquoGuard-Möller Medical GmbH, Fulda, Germany).

**Figure 2:**
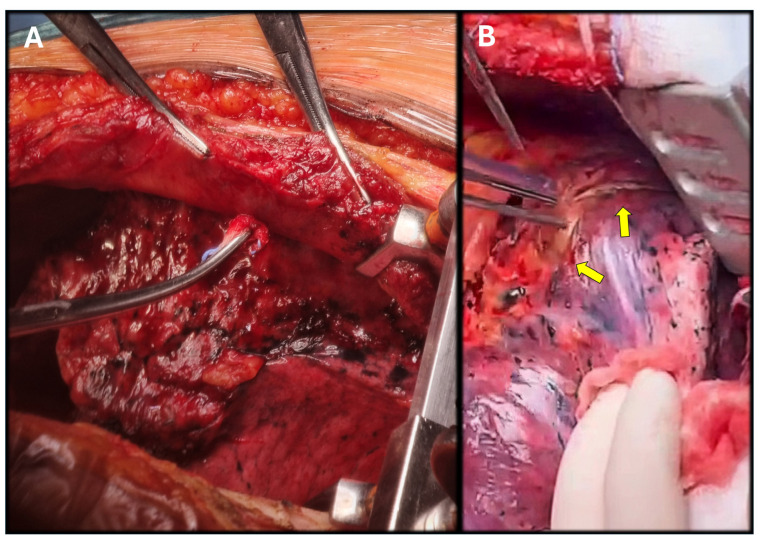
Pulmonary adhesions were carefully separated with blunt dissection during the redo thoracotomy (**A**). The pseudoaneurysm was identified in intimal contact with the visceral pleura of the left superior pulmonary lobe ((**B**), yellow arrows). Due to this, dissection during surgery was considered unsafe and clamping sites were found proximal and distal to the pseudoaneurysm.

After a left inguinal cutdown, a 5th left intercostal space redo thoracotomy was carried out, identifying tenacious pulmonary adhesions to the parietal pleura (Figure 2A). While attempting to separate the lung from the pseudoaneurysm, the risk of rupture was evident (Figure 2B); therefore, dissection was carried out proximally and distally at the clamping sites. Progressive isolation of the thoracic aorta, mobilization of the distal arch and the LSA, and isolation of the aortic arch were performed. Cardiopulmonary bypass (CPBP) with deep hypothermic circulatory arrest (DHCA) was considered as a bailout strategy if clamping could not be performed [10].

**Figure 3:**
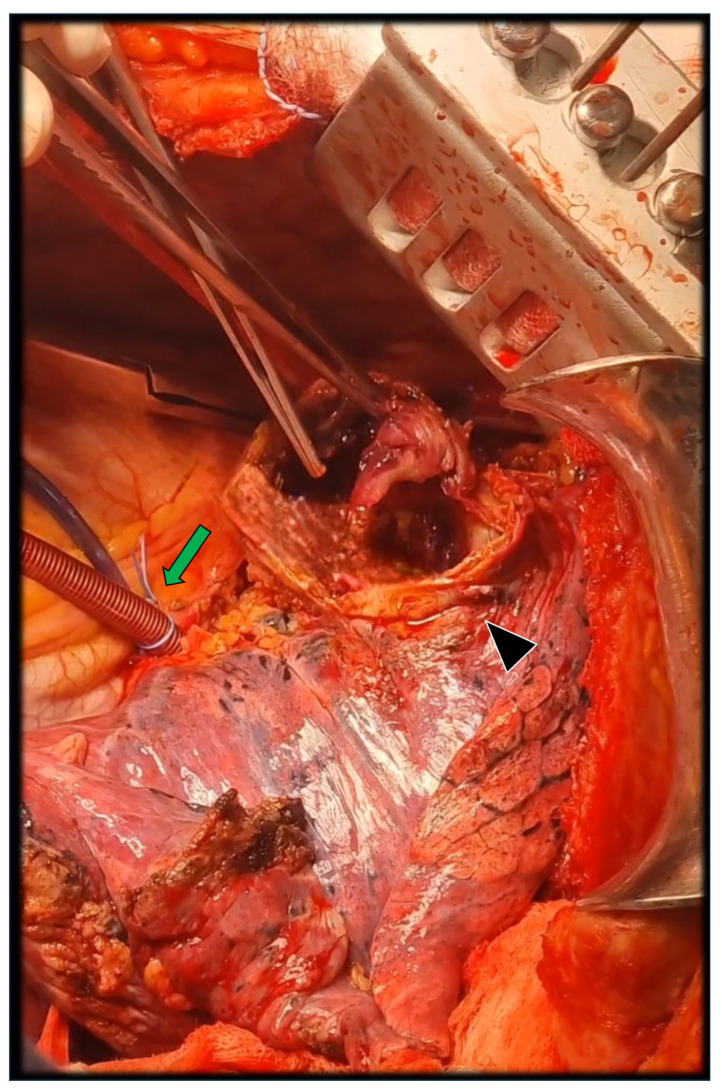
Once the left superior pulmonary vein was cannulated (green arrow) and connected to the left common femoral artery, the LHBP circuit was initiated, allowing secure and protected vascular control of the distal aortic arch, LSA and distal thoracic aorta. After systemic heparinization and clamping, the pseudoaneurysm was opened, where the intimate relation between the pseudoaneurysm’s anterior wall and the lung (black arrowhead) was evidenced. Subsequently, aortotomy was completed up to the distal aortic arch, excising the LSA at its origin.

During dissection, it was possible to achieve a proximal clamping site distal to the left common carotid artery, and accordingly, an LHBP was able to be used. Under systemic heparinization (70 IU/kg), the pericardium was incised, and the inflow cannula was placed in the left atrium through the left superior pulmonary vein (Figure 3). The out-flow cannula was positioned downstream of the distal clamp through the previously prepared left common femoral artery.

**Figure 4:**
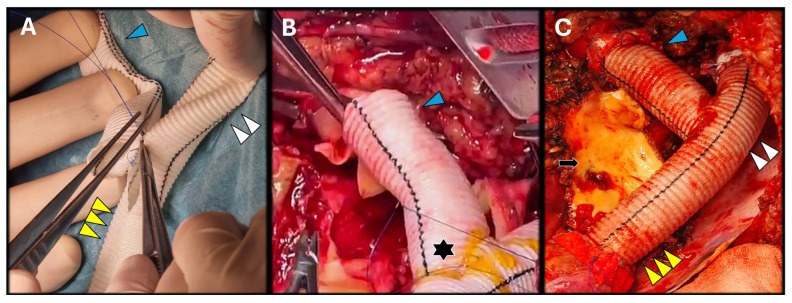
Side table preparation of a “Y” graft Dacron graft for the aortic reconstruction (Hemashield platinum 16 mm) (**A**). After arterial suture sites were prepared, distal aortic arch anastomosis was performed with 4/0 polypropylene ((**A**–**C**), blue arrowhead) and the distal thoracic aorta ((**A**,**C**), yellow triple arrowhead) with 3/0 polypropylene, both end-to-end continuous sutures with Teflon-felt reinforcement. The graft’s anastomosis was reinforced with fibrin glue ((**B**), black asterisk) for local hemostasis optimization. Once aortic anastomoses were completed, revascularization of the LSA to the “Y” graft’s branch ((**A**,**C**), double white arrowhead) was completed in the same fashion (4/0 polypropylene), concluding the distal aortic arch reconstruction (**C**). Portions of the pseudoaneurysm that were technically inseparable from the pulmonary tissue were left in situ ((**C**), black arrow) to mitigate the risk of rupture, further lung tissue damage and potential postoperative respiratory complications.

Once the distal aortic arch, the LSA, and the distal thoracic aorta were clamped, the LSA was excised, and a transection of the aorta at the distal aortic arch was performed. Then, the pseudoaneurysm was opened (Figure 3) and, through a longitudinal aortotomy (from the distal thoracic aorta up to the transected arch), the luminal side of the pseudoaneurysm was identified. Meanwhile, on a side table, a Y-graft was prepared, including a branch for the LSA (Hemashield Platinum 16 mm) with an end-to-side anastomosis (Figure 4A).

Resection of the patch was followed by the distal aortic arch reconstruction with the Y-graft. After proximal and distal end-to-end aortic anastomoses, respectively, with a 4/0 polypropylene and 3/0 polypropylene (both with Teflon reinforcement) the LSA was revascularized to the Y-graft’s branch (Figure 4B,C). The total length of the distal aortic arch reconstruction was 16 cm (distance between both aortic anastomosis) and a 3 cm length branch for the LSA. Throughout the entire surgical time, there were no signs of decreased activity on the SMEP monitoring or any pressure increase in the CSF drainage that required any specific actions. The total time of the LHBP was 55 min with an aortic cross-clamping time of 45 min. Total time from aortic-cross clamping until LSA revascularization was 60 min.

**Figure 5:**
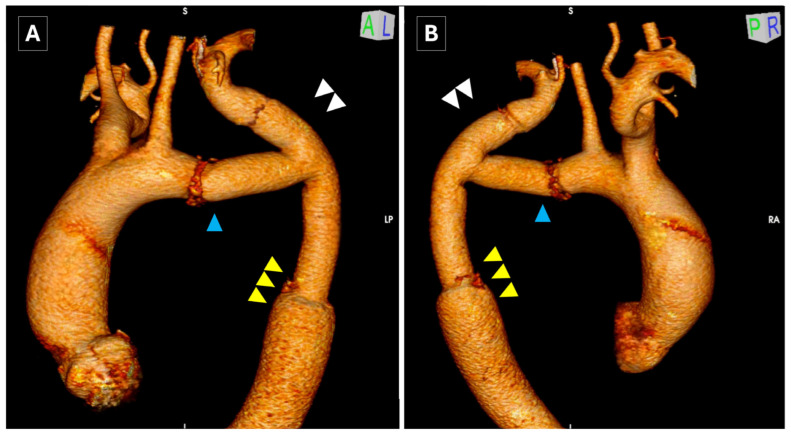
Three-dimensional CTA reconstruction at 6 months ((**A**): anterior view; (**B**): posterior view), showing the distal aortic arch reconstruction (blue arrowhead), the patency of the LSA (white double arrowhead) and the distal thoracic aorta (yellow triple arrow).

Once the LHBP was discontinued, and the cannulas removed, resolution of the preoperative pressure gradient was confirmed intraoperatively through the right femoral arterial line (right arterial line 122/69 mm Hg; right femoral line 119/65 mm Hg; mean arterial pressure gradient 3 mm Hg). Pulmonary aerostasis was verified, and closure of the thoracic wall was performed with double chest tube drainage. The postoperative course was uneventful. The chest tubes were removed on postoperative day 4, and the patient was discharged on postoperative day 6 with equalization of arterial pressure between both arms (126/71 mm Hg on the right; 120/70 mm Hg on the left) with a bilateral normal ankle-brachial index (0.97 on the right; 0.99 on the left). At discharge, his medical and antihypertensive medication included aspirin (100 mg/day), furosemide (25 mg/8 h), metoprolol (50 mg/12 h), candesartan (16 mg/12 h) and lecardipin (20 mg/12 h). At 3-month follow-up, the patient is asymptomatic and has returned to his previous activities, with a CTA showing patency of the LSA and the distal aortic arch reconstruction (Figure 5), without any sign of aortic-related complications, confirmed also at the 9-month follow-up CTA.

**Figure 6:**
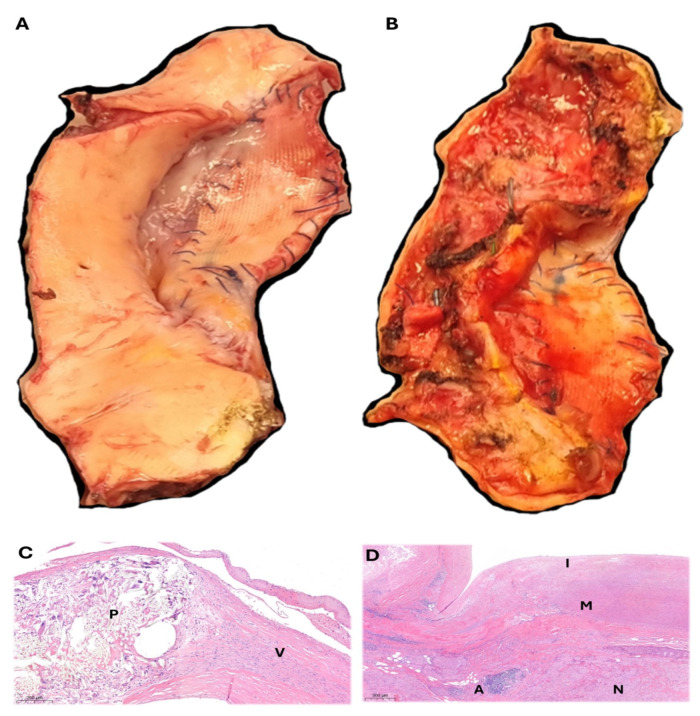
Resected segments of the aneurysm and the previous patch ((**A**): intimal face; (**B**): adventitial face), where it is possible to identify that the suture line remains intact, the Dacron tissue has not deteriorated, and the pseudoaneurysm involves mainly the surrounding aortic tissue. The histological analysis of the resected patch and adjacent aortic tissue is provided. On panel (**C**) (hematoxylin-eosin stain), the interface patch (P) and vessel’s wall (V) is illustrated. On Panel (**D**) (hematoxylin-eosin stain), chronic non-inflammatory changes are evident in the intimal (I), medial (M), and adventitial (A) layers of the native aortic wall. The presence of nerve proliferation (N) within the adventitia is consistent with chronic pathological remodeling. Moreover, marked structural alterations, including parietal fragility and pronounced fiber disarray, highlight a severely compromised and diseased vascular wall. These findings are indicative of an advanced degenerative process, consistent with pathologically altered tissue that is progressively weakening and transitioning toward an aneurysmal state.

The histopathological analysis of the removed aortic tissue and patch (Figure 6A,B) showed signs of chronic degenerative changes, including fibrosis, inflammation with immune cells, and foreign body-type reactions near the patch, which did not present signs of fiber degradation (Figure 6C). The intimal layer had fibro-cellular buildup and cholesterol deposits, while the medial layer exhibited elastic fiber fragmentation, mucoid matrix accumulation, and smooth muscle cell loss. The adventitia was fibrotic with prominent nerve structure, overall suggesting a moderate, non-inflammatory degenerative disease of the aorta with mild atherosclerosis (Figure 6D). No signs of infection were found.

## 3. Comprehensive Literature Review

CoA is a congenital arteriopathy characterized by narrowing, typically at the juxtaductal isthmus; more complex variants involve the aortic arch or thoracoabdominal aorta [11]. It often coexists with systemic vascular anomalies such as aberrant arch branching, intracranial aneurysms, and BAV—present in up to 50% of patients—which may increase the risk of ascending aortic aneurysm and dissection and requires lifelong surveillance [12,13]. However, some recent studies suggest that the coexistence of CoA and BAV is not associated with this elevated risk of aortic events [14]. Clinical presentation varies with age: neonates often present with heart failure, while older patients develop systemic hypertension [12,13]. Delayed intervention increases aneurysm risk adjacent to repair sites. Prognosis relies on prompt diagnosis, individualized treatment, and structured long-term follow-up [11,12,13].

Surgery remains the first-line in neonates and complex anatomies, with end-to-end anastomosis preferred for discrete lesions, while subclavian flap aortoplasty is an alternative in small infants, though it carries a risk of limb hypoperfusion [15]. Patch aortoplasty, especially with Dacron, is now rarely used due to high aneurysm rates [16]. Complex arch involvement or associated cardiac defects may require sternotomy with CPBP [17].

Endovascular treatment is chosen in older children and adults with suitable anatomy. Balloon angioplasty reduces gradients but carries risks of dissection and aneurysm formation [18]. Bare-metal stents improve patency and reduce recoil, while covered stents are used to reinforce fragile or aneurysm-prone segments [19].

Imaging surveillance is paramount. Due to lifelong surveillance recommendations, magnetic resonance imaging (MRI) is preferred for routine follow-up (to reduce exposure to ionizing radiation and iodinated contrast media), while CTA is reserved for patients with stents or complex anatomy. Surveillance intervals should be tailored to the type of repair, with intensified monitoring post-stenting. Strict blood pressure control and screening for BAV and cerebral aneurysms are mandatory [12,13,19].

### 3.1. Post-Aortic Coarctation Aneurysms (pCoAA)

Aneurysmal degeneration is a late complication, with incidence depending on repair type: ~40% after patch aortoplasty, 3–6% post end-to-end anastomosis, and 4–12% following endovascular repair [4,6,7,17,20]. Covered stents reduce but do not eliminate this risk. Aneurysm formation results from hemodynamic stress, patch–native tissue mismatch, and medial degeneration, while pseudoaneurysms arise from suture or patch failure and true aneurysms involve full-thickness dilation, often remaining asymptomatic until rupture [4,6,7,16,20].

A retrospective multicenter study from high-volume aortic centers [5] reported a total of 74 patients (46 male; median age 44) treated for late pCoAA. In this study, both TEVAR (n = 46) and OSR (n = 28) were suitable approaches, illustrating that OSR may offer a more definitive correction but with higher morbidity (20–30%) and mortality (3.6%) rates. TEVAR achieved a 92.5% technical success rate with lower perioperative mortality (2.2%) and faster recovery, while presenting a 4–15% rate of endoleak development during follow-up, with no reported events of spinal cord ischemia. Hybrid strategies, including carotid–subclavian bypass and TEVAR, expand the options for arch involvement, and both approaches yield comparable mid-term outcomes when tailored to anatomy and risk [5,21]. Although the optimal timing and size for treatment are currently unclear, intervention for late pCoAAs is suggested when they reach a 4.5 cm diameter [6], develop associated symptoms, or—regardless of their dimensions—present with a saccular or pseudoaneurysmal configuration at diagnosis [5].

### 3.2. Recoarctation

Recoarctation mainly affects neonates and low-birth-weight infants, with reintervention rates up to 50%, though these decrease to 5–20% in older patients. Risk factors include early repair, arch hypoplasia, residual gradients, BAV and syndromes (such as Turner). Pathogenesis involves residual ductal tissue, fibrosis, neointimal proliferation, and growth mismatch, while endothelial injury from prior interventions worsens restenosis [13,22].

Clinically, recoarctation may be asymptomatic or present with upper-limb hypertension, radiofemoral delay, or claudication. Doppler echocardiography screens for it, with gradients >20 mmHg indicating hemodynamic significance. MRI and CTA provide anatomical detail, while catheterization remains the gold standard for functional assessment [11,13,22].

Therapy depends on age and anatomy. Balloon angioplasty is preferred in infants and young children despite a 10–30% restenosis risk. In patients >25–30 kg, endovascular stenting offers more durable results. Covered stents are chosen in aneurysm-prone anatomy and allow staged redilation to accommodate patient growth, whereas surgery is reserved for complex or failed endovascular cases, offering definitive repair at higher operative risk [10,15,16].

At long-term follow-up, up to 40% develop persistent hypertension, increasing the risk of ventricular hypertrophy, aortic aneurysm, and premature coronary artery disease. Optimal outcomes require precise repair, structured imaging, and comprehensive cardiovascular risk management [11,12,13].

Several authors previously reported inliterature that the progression of late complications after CoA repair, mainly pCoAA, appears to be significantly more aggressive than that of atherosclerotic ones, irrespective of maximal diameter, with high rate of rupture if left untreated. Given the high risk of rupture and associated mortality, guidelines from the American Heart Association and the American College of Cardiology recommend routine surveillance of the repair site using cross-sectional imaging—MRI or CTA—at a minimum interval of every five years in adults with congenital heart disease [5].

## 4. Discussion

Several authors suggest that the natural progression of a late post-coarctectomy aneurysm (or pseudoaneurysm) appears more malignant than an atherosclerotic one, regardless of the maximum diameter [5]. Therefore, upon identification, prompt resolution is advisable.

A wide range of surgical strategies have been described in the literature to address aortic coarctation [3], its recurrence, and complications derived from its initial correction [5,23,24]. Described treatment strategies include OSR [25], hybrid [26] and TEVAR [27], depending on the clinical presentation, the patient’s perioperative risk, anatomical features and the center’s experience. According to the literature, both OSR and TEVAR are safe and feasible, with good early and mid-term results [5].

Due to the anatomical features of our patient (hypoplastic proximal aortic arch; recoarctation; LSA hypertrophy and origin involvement; no proximal landing zone; >50% mismatch between arch and distal thoracic aorta diameters), a standard TEVAR was discarded. Embolization of the LSA [28] could have allowed extension for a TEVAR in zone 2, but guidelines do not recommend occlusion or embolization of the LSA unless in an emergent situation due to the potential risk of stroke and spinal cord ischemia [29]. Furthermore, the hypertrophic LSA (18 mm) could have increased the risk of a type II endoleak and long-term reintervention at this level [30].

To address this, debranching of supra-aortic vessels has been widely used to extend the proximal landing zone into the proximal aortic arch [31] while preserving their patency. A left carotid–subclavian bypass (or LSA transposition) may have been used with a proximal landing site in zone 2 [21]. Despite the excellent patency rates of the carotid–subclavian bypass (97.6% primary patency at 2 years) [31], early postoperative complications can occur in up to 23% of patients, with bleeding and peripheral nerve injury being the most common [31]. Extension to a zone 1 TEVAR could have provided an additional 20 mm neck length but at the expense of performing a carotid–carotid bypass in addition to a left carotid–subclavian bypass. This approach was discarded due to the higher mortality and stroke rates reported for this surgical combination (carotid–carotid + carotid–subclavian bypass + TEVAR) compared to only carotid–subclavian bypass + TEVAR [32]. Carotid–carotid transposition has also been described as a feasible debranching technique with good short-term results [33], but its use is not widely adopted.

To preserve the LSA and supra-aortic vessels in a total endovascular fashion, some devices may include branches of fenestrations intended to maintain its patency, with good mid-term results when compared to surgical debranching [34,35].

One of the technical difficulties of an endovascular approach in our patient was the diameter mismatch (>50%) between the proximal aortic arch (zones 1 and 2) and the distal thoracic aorta. This feature might have required a tapered custom-made device (CMD) to match the diameter mismatch, delaying the intervention due to the manufacturing time of a CMD. Although the reported rate of aortic aneurysm rupture while awaiting a CMD is low [36], the “urgent” characteristics of our patient’s lesion (pseudoaneurysm) led us to abandon this option. In situ fenestration, parallel grafting and physician-modified endografts were also discarded since these techniques are considered off-label and outside the instructions of use, and although our case required prompt resolution, it was not an emergent situation.

Another, and perhaps the most important, factor that led us away from an endovascular approach was the recoarctation. This might have resulted in inadequate or insufficient expansion of a self-expandable thoracic endograft, increasing the risk of endoleaks and subsequent sustained patency of the pseudoaneurysm and risk of rupture. Balloon-expandable covered aortic stents have also been used for recoarctation scenarios [23] but performing angioplasty in friable and unstructured aortic tissue could have been a risky intervention, increasing the chance of intraoperative rupture.

Kudo et al. [37] evaluated the long-term outcomes of a hybrid approach using proximal landing zones 0, 1, and 2 for aortic arch pathologies over 12 years at a single center with 348 patients. The procedures combined various surgical debranching techniques—not including carotid–carotid nor carotid–subclavian bypass—with TEVAR to reduce invasiveness compared to open arch repair. Results showed low 30-day mortality (0.6%) and stroke rate (1.1%), with 10-year survival and aorta-related death-free rates of 75% and 97.2%, respectively. Nonetheless, 135 patients (38.8%) still required a median sternotomy and 94 (27%) underwent CPBP. Other hybrid techniques, like an ascending to descending aorta bypass and antegrade stent-graft delivery into the LSA [17] have been described sporadically. The frozen elephant technique has also been reported as a feasible option to treat aortic coarctation and late pCoAA [5,38]. Since our patient did not present involvement of the ascending aorta or proximal arch, a total or hybrid arch repair—via sternotomy with CPBP—was discarded.

OSR through redo thoracotomy carries an intrinsically higher risk of intra- and postoperative complications in both non-aortic [39] and aortic-related interventions [40]. The most frequent complications associated with redo thoracotomy performed during aortic surgery are respiratory failure and bleeding requiring re-exploration. In addition, Baar et al. [39] described that age (OR = 1.02 per year increase) and an LVEF < 60% are both independent, significant risk factors for developing postoperative complications after thoracotomy. Respiratory complications, including prolonged ventilation and pneumonia, are particularly common due to adhesions and lung injury during re-entry, with reported rates of 15–22% in large series of redo thoracoabdominal aortic repairs. Bleeding is also a major risk, often necessitating reoperation, and is exacerbated by difficult dissection through scar tissue [40,41]. Another contributing factor is the fragility of the aortic tissue itself, aortic clamping near the left common carotid artery, and the involvement of other structures such as the phrenic and vagus nerves [5]. Despite this challenging approach, previous published series indicate that OSR via redo thoracotomy with LHBP is feasible and can be performed with acceptable morbidity and mortality at experienced centers [42]. In a consecutive series of 60 patients by undergoing redo thoracotomy for descending or thoracoabdominal aortic repair, Etz et al. [40] reports that LHBP was associated with a lower rate of adverse outcomes compared to clamp-and-sew or partial CPBP.

Although the “diseased” segment—which included the pseudoaneurysm and recoarctation—of the distal aortic arch and thoracic aorta measured approximately 8 cm in length (from zone 2 to zone 3, according to the preoperative CTA reconstruction and measurements), the total length of the interposition graft was approximately 16 cm (from the proximal anastomosis in zone 2 to the distal anastomosis in zones 3–4). This increase in interposition length is explained by the intraoperative identification of non-friable, healthy aortic tissue—which can be difficult to assess on the preoperative CTA—necessary to securely cross-clamp and perform the aortic anastomoses. In addition, due to excision of the previous patch and partial removal of the pseudoaneurysm walls, along with transection of the LSA for later revascularization, the configuration and distance measurements were adjusted to accommodate these intraoperative anatomical changes. If the aortic clamping sites become inaccessible or unsafe, the complexity of the surgery increases, sometimes requiring CPBP [27,37,38] or DHCA [10,43]. In our case, prior control of the femoral vein was also obtained as a preventive measure until a proximal aortic clamping site was identified.

The risk of spinal cord ischemia during the aortic cross-clamping period (45 min) was mitigated by the intraoperative strategy, which included distal aortic perfusion via the LHBP [8,9] and the selection of the distal aortic clamp site. The clamp was positioned proximally to the level of the 8th–9th thoracic vertebrae, enabling perfusion of the distal intercostal arteries through the LHBP, which are known to play a major role in supplying arterial perfusion to the spinal cord through the great anterior segmental medullary artery (or Adamkiewicz artery) [44]. Furthermore, after the aortic clamp was released—restoring antegrade pulsatile aortic perfusion—the LSA was revascularized via the “Y” graft, preserving the collateral spinal cord perfusion originating from the LSA [44].

Based on the above, careful preoperative planning and risk stratification are critical for selecting the optimal surgical strategy. While an endovascular solution may offer a less invasive approach (particularly appealing in a redo case) [26,45,46], in our case, due to the patient’s age and absence of contraindications to sustain OSR (LVEF > 60%), combined with the known reintervention rates of complex endovascular aortic procedures [47], the redo-thoracotomy approach and distal aortic arch reconstruction with a “Y” graft interposition—to preserve the LSA—under LHBP (to provide distal aortic perfusion during aortic cross-clamping) allowed us to perform a successful aortic arch repair without the need for sternotomy, CPB, or cervical vessel debranching and reducing the risk of spinal cord ischemia with good short- and mid-term outcomes.

The histological findings of the resected aortic tissue of our patient correlates with previous similar reports in the literature [48,49], where the synthetic patch remains mostly unaltered and the affected aortic tissue around the repair is the part that develops the degeneration [48,49], reinforcing the need for life-long surveillance of these patients [5,12,13].

## 5. Conclusions

Open surgical repair for post-coarctectomy pseudoaneurysm is a viable therapeutic option. Redo open surgery, despite its challenges, can provide a more definitive solution—particularly for fit and young patients without contraindications for OSR—while reserving the endovascular approach for high-risk patients or more favorable anatomy.

For patients with complex anatomy, concurrent pseudoaneurysm and recurrent coarctation who are unsuitable for endovascular repair, redo open surgery is a safe and effective option in experienced teams. The treatment choice should be tailored individually, since the long-term durability of stent grafts in young patients is still unknown. High-volume centers with experience in complex aortic disease might offer the best results for these patients.

## 6. Future Directions and Perspective

Advancing the understanding of the molecular, genetic and clinical mechanisms of CoA, recoarctation and aneurismatic degeneration [50,51], and other associated conditions [52,53], may facilitate the development of targeted therapies aimed at reducing recurrence and late complication rates. In parallel, future research paths could investigate the follow-up strategy, the optimal timing of intervention and the long-term effectiveness of the repair strategies [54]. Additionally, comprehensive data from multicenter registries and real-world clinical settings, combined with artificial intelligence assistance [55] will be critical in shaping evidence-based management protocols and guiding clinical decision-making in future practice.

## Data Availability

Data unavailable for consultation due to privacy restrictions.

## Abbreviations

The following abbreviations are used in this manuscript:

CoA	Aortic coarctation
pCoAA	Post-aortic coarctation aneurysm
LVEF	Left ventricular ejection fraction
LSA	Left subclavian artery
OSR	Open surgical repair
BAV	Bicuspid aortic valve
SMEP	Somatosensory-evoked potentials
CSF	Cerebral–spinal fluid
CTA	Computerized tomography angiography
MRI	Magnetic resonance imaging
CPBP	Cardiopulmonary bypass
DHCA	Deep hypothermic circulatory arrest
LHBP	Left heart bypass
TEVAR	Thoracic endovascular aortic repair
CMD	Custom-made device
